# Supplementary material for: NMR-Solver: automated structure elucidation via large-scale spectral matching and physics-guided fragment optimization
Source: Nat Commun. 2026 Apr 2;17:4740. doi: 10.1038/s41467-026-71315-0 (PMC13216333; doi:10.1038/s41467-026-71315-0)
Supplement: Supplementary file 1 — Supplementary Information [file 41467_2026_71315_MOESM1_ESM.pdf]

Supplementary Information for "NMR-Solver: Automated Structure Elucidation via Large-Scale Spectral Matching and Physics-Guided Fragment Optimization"

Yongqi Jin[1,2], Jun-Jie Wang[2,3], Fanjie Xu[2,4], Xiaohong Ji[2], Zhifeng Gao[2], Linfeng Zhang[2,5], Guolin Ke[2*], Rong Zhu[3,5*], Weinan E[1,5,6*]

[1]School of Mathematical Sciences, Peking University, Beijing, 100871, China.
[2]DP Technology, Beijing, 100080, China.
[3]College of Chemistry and Molecular Engineering, Peking University, Beijing, 100871, China.
[4]Institute of Artificial Intelligence, Xiamen University, Xiamen, 361005, China.
[5]AI for Science Institute, Beijing, 100080, China.
[6]Center for Machine Learning Research, Peking University, Beijing, 100871, China.

*Corresponding author(s). E-mail(s): kegl@dp.tech; rongzhu@pku.edu.cn; weinan@math.pku.edu.cn;
Contributing authors: yongqijin@stu.pku.edu.cn; junjie-wang@pku.edu.cn; xufanjie@stu.xmu.edu.cn; jixh@dp.tech; gaozf@dp.tech; zhanglf@dp.tech;

Contents

Supplementary Notes 4

Supplementary Note 1. Evaluation Metrics . . . 4
Supplementary Note 2. Ablation Study . . . 5
Supplementary Note 3. Incorporation of Multiplicity Patterns in the Set Similarity . . . 6
Supplementary Note 4. Comparison of Spectral Similarity Metrics . . . 7
Supplementary Note 5. Algorithm for Fragment-NMR-Based Molecular Optimization . . . 8
Supplementary Note 6. Algorithm Execution for Product Prediction Cases . 9
Supplementary Note 7. General Experimental Methods . . . 10
Supplementary Note 8. Experimental Procedures and Characterization for the Reaction in Fig. 3a . . . 11
Supplementary Note 9. Experimental Procedures and Characterization for the Reaction in Fig. 3b . . . 12
Supplementary Note 10. Experimental Procedures and Characterization for the Reaction in Fig. 3c . . . 13
Supplementary Note 11. Experimental Procedures and Characterization for the Reaction in Fig. 3f . . . 14
Supplementary Note 12. Web App . . . 15

Supplementary Tables 16

Supplementary Table 1. Hyperparameters for similarity metrics . . . 16
Supplementary Table 2. Hyperparameters for database index . . . 17
Supplementary Table 3. Hyperparameters for FB-MO . . . 18
Supplementary Table 4. Comparison with current methods on the experimental dataset (stereochemistry ignored) . . . 19
Supplementary Table 5. Comparison with current methods on the experimental dataset (stereochemistry preserved) . . . 20
Supplementary Table 6. Comparison of NMR-Solver performance with and without reactants . . . 21
Supplementary Table 7. Ablation study on the contribution of the optimization stage . . . 22
Supplementary Table 8. Performance of spectral similarity metrics under varying noise and perturbation conditions ($p = 0$) . . . 23
Supplementary Table 9. Performance of spectral similarity metrics under varying noise and perturbation conditions ($p = 0.2$) . . . 24
Supplementary Table 10. Comparison of NMR-Solver performance with or without multiplicity information . . . 25
Supplementary Table 11. Allowed cleavage bond pairings in FB-MO . . . 26

Supplementary Figures 27

Supplementary Figure 1. Comparison of spectral similarity metrics under varying noise and perturbation conditions . . . 27

Supplementary Figure 2. Atom count distribution of the 450 molecules in the literature dataset 28
Supplementary Figure 3. Element statistics of the 450 molecules in the literature dataset 29
Supplementary Figure 4. NMR-Solver's Predicted Structures for the Case in Fig. 3a 30
Supplementary Figure 5. NMR-Solver's Predicted Structures for the Case in Fig. 3b 31
Supplementary Figure 6. NMR-Solver's Predicted Structures for the Case in Fig. 3c 32
Supplementary Figure 7. NMR-Solver's Predicted Structures for the Case in Fig. 3d 33
Supplementary Figure 8. NMR-Solver's Predicted Structures for the Case in Fig. 3e 34
Supplementary Figure 9. NMR-Solver's Predicted Structures for the Case in Fig. 3f 35
Supplementary Figure 10. ^{1}H and ^{13}C NMR spectra of ethyl 4-benzyl-3-(phenylethynyl)benzoate 36
Supplementary Figure 11. ^{1}H and ^{13}C NMR spectra of dibutyl 2-chloro-2-(7-chlorohept-5-yn-1-yl)malonate 37
Supplementary Figure 12. ^{1}H and ^{13}C NMR spectra of methyl 4-(3-methoxy-3-oxo-1-phenylpropoxy)benzoate 38
Supplementary Figure 13. ^{1}H and ^{13}C NMR spectra of methyl 6,7-diethyl-4,4-dimethyl-2-oxobicyclo[3.2.0]hept-6-ene-1-carboxylate 39
Supplementary Figure 14. ^{1}H-^{13}C HMBC and HSQC spectra of ethyl 4-benzyl-3-(phenylethynyl)benzoate 40

Supplementary Notes

Supplementary Note 1. Evaluation Metrics

To quantitatively evaluate the performance of molecular structure prediction from NMR spectra, two complementary metrics are employed: **Recall** and **Tanimoto similarity**.

Recall@K measures the proportion of test cases where the correct molecular structure appears among the top-K predicted candidates. Specifically, for a given sample, success is defined as the ground-truth molecular structure being ranked within the top-K predictions. Recall@K is computed as the average success rate across all test samples:

$$\text{Recall@K} = \frac{1}{N}\sum_{i=1}^{N} \mathbb{I}\left(\text{target}_i \in \text{top-}K \text{ predictions}_i\right),$$

where $\mathbb{I}(\cdot)$ is the indicator function that returns 1 when the condition is satisfied and 0 otherwise.

In addition to exact structure matching, **Tanimoto similarity** [1, 2] is used to measure the molecular similarity between the ground-truth and predicted structures based on their Morgan fingerprints [3, 4]. In this work, Morgan fingerprints are computed using the RDKit toolkit as 2048-bit vectors with a radius of $r = 2$, capturing the local structural features of each molecule. The Tanimoto coefficient is defined as:

$$\text{Tanimoto}(A, B) = \frac{|A \cap B|}{|A \cup B|},$$

where A and B are the fingerprint bit vectors of the two molecules. A higher Tanimoto score indicates greater structural similarity.

We also define **Tanimoto@K** to measure the maximum Tanimoto similarity between the ground-truth molecule and any of the top-K predicted structures. This metric provides insight into how structurally similar the best-ranked prediction is to the true molecule, even if an exact match is not achieved. Formally:

$$\text{Tanimoto@K} = \frac{1}{N}\sum_{i=1}^{N} \max_{j=1,\ldots,K} \Big(\text{Tanimoto}\big(f_{\text{Morgan}}(\text{target}_i), f_{\text{Morgan}}(\text{prediction}_{i,j})\big)\Big)$$

Both metrics are reported across multiple values of K (e.g., $K = 1, 3, 10$) to provide a comprehensive view of the model's performance in terms of both exact structure retrieval and structural closeness to the ground truth.

Supplementary Note 2. Ablation Study

To independently evaluate the contribution of the optimization stage, an ablation study is conducted by removing target molecules from the initial retrieval results, forming a contrast with the default process. This ensures that any remaining performance improvements are attributed to the optimization process refining non-trivial candidates, rather than retrieval recall alone.

The experimental results, shown in Supplementary Table 7, demonstrate that the method exhibits only a marginal performance drop (less than 2% across all settings for both ^{1}H and ^{13}C NMR). This indicates that the optimization module retains its effectiveness even when handling molecules not present in the database. Such a small drop suggests that the optimization process is the primary driver of the improvements, rather than relying heavily on retrieval recall.

Through this ablation study, the robustness of the optimization process is underscored. Furthermore, the results show that NMR-Solver is capable of performing NMR analysis for molecules previously unknown to humans, further demonstrating its generalization ability.

Supplementary Note 3. Incorporation of Multiplicity Patterns in the Set Similarity

For experimental ^{1}H NMR spectra, multiplicity patterns are typically reported in the spectral data and are directly extracted. For simulated spectra, multiplicity patterns are estimated based on the first-order coupling effects, which predicts the splitting pattern from the number of neighboring protons in the molecular structure.

The multiplicity patterns are incorporated into the matching function as multiplicative weights, ω_{ij}, which modifies the original scoring function. This additional information helps to refine the peak assignments and improve the overall similarity score.

Details of the exact implementation are as follows:

$$S(\mathcal{X}, \mathcal{Y}) = \frac{1}{\sqrt{mn}} \max_{P \in \mathcal{P}} \sum_{(i,j) \in P} \omega_{ij} f(x_i, y_j),$$

where ω_{ij} is set to w_1 (default value: 1.0) if the multiplicity pattern of x_i matches that of y_j, and to w_2 (default value: 0.8) otherwise.

A comparison between using multiplicity patterns in the ^{1}H NMR spectra and using chemical shifts alone is provided in Supplementary Table 10. Incorporating multiplicity yields a modest yet consistent improvement. Multiplicity and J-coupling information are expected to offer further gains for NMR-based structure elucidation as more accurate prediction methods become available.

Supplementary Note 4. Comparison of Spectral Similarity Metrics

Within the NMR-Solver framework, we employ two complementary similarity scoring methods: **vector similarity** and **set similarity**. To demonstrate their effectiveness and robustness in capturing structural and spectral correspondence, these metrics are compared against conventional approaches such as the **Wasserstein distance** [5], which has been widely used for spectrum matching in chemical informatics.

The performance of both vector similarity and set similarity is evaluated against the Wasserstein distance on a test set of 1000 randomly selected molecules from our NMR database. For each query molecule, a set of 1000 candidate molecules is retrieved using vector index. The goal is to identify the correct molecule from this set of candidates. To simulate realistic experimental variations, Gaussian-distributed chemical shift perturbations were applied to the query spectra, along with random peak deletions and insertions to model signal overlap and impurity effects.

The results (Supplementary Figure 1) demonstrate that the set similarity measure exhibits strong robustness against both spectral shift noise and peak deletion/insertion perturbations, significantly outperforming the alternative methods under realistic degradation conditions. In contrast, the vector similarity method degrades rapidly with increasing noise levels (Supplementary Figure 1a), reflecting its poor resilience to large deviations between predicted and experimental chemical shifts. The Wasserstein distance performs well under shift noise but deteriorates noticeably as the probability of random peak deletion or insertion increases (Supplementary Figure 1b), highlighting its vulnerability to peak overlap and spurious peaks. Detailed experimental results are provided in Supplementary Tables 8 and 9.

This comparative analysis underscores the practical advantage of the set similarity as a scoring function in real-world applications, where spectral data are often incomplete or noisy. Moreover, it enables the robust identification of molecules with partial spectral overlap, supporting queries for compounds that share chemically meaningful fragments—such as functional groups or ring systems—even in the absence of full structural similarity.

Supplementary Note 5. Algorithm for Fragment-NMR-Based Molecular Optimization

We provide a pseudocode description of the Fragment-NMR-Based Molecular Optimization (FB-MO) algorithm.

Algorithm 1 Fragment-NMR-Based Molecular Optimization (FB-MO)

Input: Chemical shift lists X_{H} and X_{C}
Parameters: N_{iter}, N_{pool}, N_{frag}, N_{pair}, N_{mol}, N_{topk}
$X_0 = \{X_{\mathrm{H}}; X_{\mathrm{C}}\}$
$\mathbf{v}_0 \leftarrow \mathrm{VectorEncoding}(X_0)$
$\mathcal{M} \leftarrow \mathrm{DatabaseSearch}(X_0,\ N_{\mathrm{pool}})$
for $i = 1$ **to** N_{iter} **do**
  $\mathcal{F} \leftarrow \cup_{m\in\mathcal{M}}\mathrm{FragmentMolecule}(m)$
  for each fragment $f \in \mathcal{F}$ **do**
    $X_{\mathrm{f}} \leftarrow \mathrm{InheritChemicalShifts}(f)$ ▷ Estimate by inherited chemical shifts
    $\mathbf{v}_f \leftarrow \mathrm{VectorEncoding}(X_{\mathrm{f}})$
  end for
  for each cut type t **do**
    $\mathcal{I}_t \leftarrow \mathrm{CreateVectorIndex}(\{v_f\}_{f\in\mathcal{F}_t})$
  end for
  $\mathcal{P} \leftarrow \emptyset$
  for each fragment $f \in \mathcal{F}$ **do**
    $\mathbf{v}_{\mathrm{comp}} \leftarrow \mathbf{v}_0 - \mathbf{v}_f$
    $\mathcal{C}_f \leftarrow \mathrm{ANNSearch}(\mathbf{v}_{\mathrm{comp}},\ \mathcal{I}_t,\ N_{\mathrm{frag}})$ ▷ Filter by vector index
    $\mathcal{P} \leftarrow \mathcal{P} \cup \{(f, c) : c \in \mathcal{C}_f\}$
  end for
  $\mathcal{P} \leftarrow \mathrm{SelectTop}(\mathcal{P}, N_{\mathrm{pair}}, \|\mathbf{v}_0 - \mathbf{v}_{f_1} - \mathbf{v}_{f_2}\|)$ ▷ Filter by vector similarity
  $\mathcal{P} \leftarrow \mathrm{SelectTop}(\mathcal{P}, N_{\mathrm{mol}}, \mathrm{SetSimilarity}(X_0, X_{f_1} \cup X_{f_2}))$
  ▷ Filter by set similarity
  $\mathcal{M}_{\mathrm{new}} \leftarrow \{\mathrm{CombineFragments}(p) : p \in \mathcal{P}\}$
  $X_m \leftarrow \mathrm{ForwardPredictionModel}(m)$ for each $m \in \mathcal{M}_{\mathrm{new}}$
  ▷ Re-estimate by forward model
  $\mathcal{M} \leftarrow \mathcal{M} \cup \mathcal{M}_{\mathrm{new}}$
  $\mathcal{M} \leftarrow \mathrm{SelectTop}(\mathcal{M}, N_{\mathrm{pool}}, \mathrm{SetSimilarity}(X_0, X_m))$
  ▷ Filter by set similarity
  if $\mathrm{TopK}(\mathcal{M},\ N_{\mathrm{topk}})$ unchanged from previous iteration **then**
    break
  end if
end for
return $\mathrm{TopK}(\mathcal{M},\ N_{\mathrm{topk}})$
Output: Top-N_{topk} candidate molecules

Supplementary Note 6. Algorithm Execution for Product Prediction Cases

The algorithm was run using its default parameters (see in Supplementary Table 3). Reactants were included as additional candidates in the initial molecular pool. Permissible elements were restricted to the organic elements present in the reactants and reactive reagents, with no constraints on molecular formula.

The experimental NMR spectra and other characterization data for the cases in Fig. 3a, 3b, 3c, and 3f are provided in the Supplementary Note 8–11, while those for Fig. 3d and 3e are reported in the publications by Fu [6] and Cheng [7], respectively. Detailed results from NMR-Solver are provided in Supplementary Figures 4–9.

Supplementary Note 7. General Experimental Methods

Chemicals: Unless otherwise noted, reagents purchased from commercial suppliers were used as received. All manipulations of air-sensitive materials were carried out in oven-dried glassware under a nitrogen atmosphere using standard Schlenk or glove-box techniques. Analytical thin-layer chromatography was conducted with glass TLC plates (HSGF254), and spots were visualized under UV light or after treatment with standard TLC stains. Flash silica gel chromatography was performed manually using silica gel (200-300 mesh) supplied by Tsingtao Haiyang or automatically on SepaBean machine T Flash Chromatography System using SepaFlash UltraPure irregular silica gel (40–63 μm, 60 Å) supplied by Santai Science. "PE" refers to petroleum ether. "EA" refers to ethyl acetate. "DCE" refers to dichloroethane. All chemicals were weighed on the bench top, in the air. All reactions were set up using standard Schlenk techniques.

NMR spectroscopy: NMR spectra were collected on a Bruker 400 MHz or a Bruker 500 MHz spectrometer at ambient temperature. chemical shifts (δ) are reported in ppm downfield from tetramethylsilane, using the solvent resonance as an internal standard. The following abbreviations were used to explain the multiplicities: s = singlet, d = doublet, t = triplet, q = quartet, m = multiplet.

HRMS: High-resolution mass data (electron spray ionization) was recorded by a Solarix XR Fourier Transform Ion Cyclotron Resonance Mass Spectrometer.

Supplementary Note 8. Experimental Procedures and Characterization for the Reaction in Fig. 3a

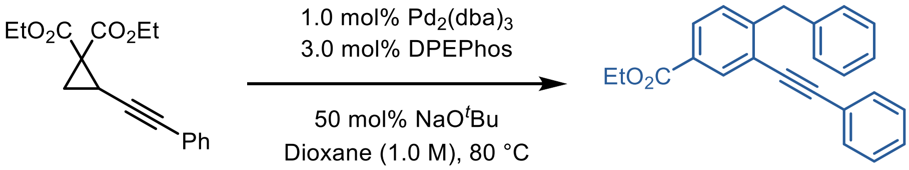

Ethyl 4-benzyl-3-(phenylethynyl)benzoate

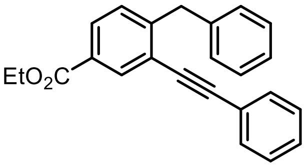

An oven-dried 10 mL re-sealable screw-cap vial equipped with a Teflon-coated magnetic stir bar was charged with Pd(dba)$_3$ (3.7 mg, 0.004 mmol, 0.010 equiv.), DPEPhos (6.5 mg, 0.012 mmol, 0.030 equiv.), and NaO^{t}Bu (19.2 mg, 0.20 mmol, 0.50 equiv.). The reaction vessel was then briefly evacuated and backfilled with nitrogen (this sequence was repeated a total of three times). Dioxane (1.0 mL) and diethyl 2-(phenylethynyl)cyclopropane-1,1-dicarboxylate (114.0 mg, 0.40 mmol, 1.0 equiv.) were added to the reaction vessel via syringe sequentially. The reaction mixture was stirred at 80 °C for 20 h. The solvents were removed *in vacuo* and the residue was purified by Al_2O_3 column chromatography (PE/ethyl acetate) to afford the title compound as a colorless oil.

^{1}H NMR (500 MHz, $CDCl_3$) δ 8.22 (d, J = 1.8 Hz, 1H), 7.91 (dd, J = 8.1, 1.8 Hz, 1H), 7.52–7.48 (m, 2H), 7.37–7.33 (m, 3H), 7.32–7.22 (m, 6H), 4.38 (q, J = 7.1 Hz, 2H), 4.29 (s, 2H), 1.40 (t, J = 7.1 Hz, 3H).
^{13}C NMR (126 MHz, $CDCl_3$) δ 165.96, 147.83, 139.73, 133.49, 131.60, 129.54, 129.42, 129.02, 128.74, 128.56, 128.55, 128.42, 126.36, 123.33, 123.02, 94.15, 87.46, 61.08, 40.42, 14.35.

HRMS (ESI): $[M + H]^+$ calculated for $C_{24}H_{21}O_2$: 341.1534, found: 341.1536.

Supplementary Note 9. Experimental Procedures and Characterization for the Reaction in Fig. 3b

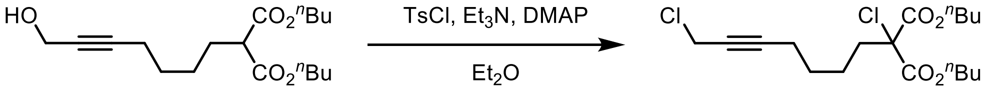

Dibutyl 2-chloro-2-(7-chlorohept-5-yn-1-yl)malonate

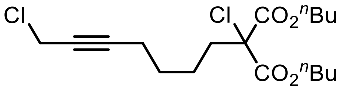

An oven-dried 100 mL Schlenk flask containing dibutyl 2-(7-hydroxyhept-5-yn-1-yl)malonate (1.00 g, 13.2 mmol, 1.0 equiv.), DMAP (0.10 g, 1.3 mmol, 0.1 equiv.) and TsCl (1.50 g, 20.0 mmol, 1.5 equiv.) was evacuated and backfilled with N_2 three times. To the flask was added Et_2O (40 mL) and cooled to 0 °C, followed by slow addition of Et_3N (4.6 mL, 2.5 mmol, 2.5 equiv.). After stirring for 30 min at 0 °C, the reaction mixture was allowed to warm gradually to r.t. and stirred until full conversion as judged by TLC. The reaction was quenched by the addition of aqueous HCl solution (1.0 M). The aqueous phase was washed with aqueous HCl solution (1.0 M) three times and the organic layer was dried over anhydrous Na_2SO_4, filtered, and concentrated *in vacuo*. The residue was purified by silica gel column chromatography (PE/ethyl acetate) to afford dibutyl 2-chloro−2-(7-chlorohept−5-yn−1-yl)malonate (176 mg, 15%) as a colorless liquid.

^{1}H NMR (400 MHz, $CDCl_3$) δ 4.21 (t, J = 6.6 Hz, 4H), 4.12 (t, J = 2.3 Hz, 2H), 2.30–2.20 (m, 4H), 1.69–1.60 (m, 4H), 1.60–1.45 (m, 4H), 1.43–1.32 (m, 4H), 0.93 (t, J = 7.4 Hz, 6H).
^{13}C NMR (101 MHz, $CDCl_3$) δ 167.01, 86.96, 75.55, 71.03, 66.91, 37.17, 31.26, 30.50, 28.09, 23.42, 19.08, 18.74, 13.73.

HRMS (ESI): $[M+Na]^+$ calculated for $C_{18}H_{28}Cl_2O_4Na$: 401.1257, found: 401.1260.

Supplementary Note 10. Experimental Procedures and Characterization for the Reaction in Fig. 3c

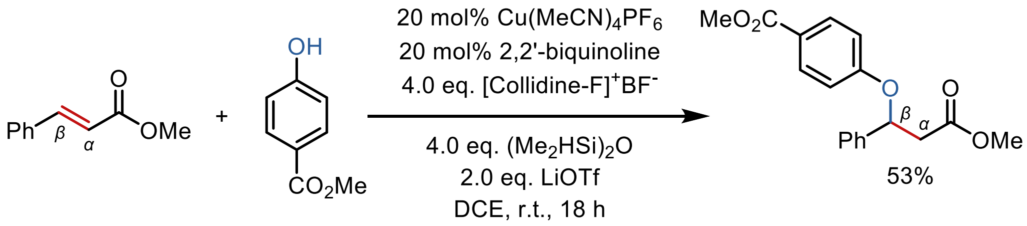

Methyl 4-(3-methoxy-3-oxo-1-phenylpropoxy)benzoate

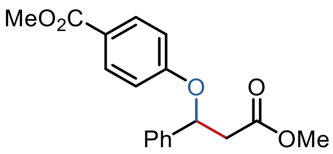

An oven-dried 10 mL re-sealable screw-cap vial equipped with a Teflon-coated magnetic stir bar was charged with methyl 4-hydroxybenzoate (91.2 mg, 0.60 mmol, 2.0 equiv.), $Cu(MeCN)_4PF_6$ (22.4 mg, 0.060 mmol, 0.20 equiv.), 2,2'-biquinoline (15.4 mg, 0.060 mmol, 0.20 equiv.), 1-fluoro-2,4,6-trimethylpyridinium tetrafluoroborate (272.4 mg, 1.20 mmol, 4.0 equiv.), and LiOTf (187.2 mg, 1.20 mmol, 2.0 equiv.). The reaction vessel was then briefly evacuated and backfilled with nitrogen (this sequence was repeated a total of three times). 1,2-Dichloroethane (3.0 mL), methyl cinnamate (48.6 mg, 0.30 mmol, 1.0 equiv.), and 1,1,3,3−tetramethyldisiloxane (160.8 mg, 1.20 mmol, 4.0 equiv.) were added to the reaction vessel via syringe sequentially. The reaction mixture was stirred at r.t. for 18 h before being diluted with Et_2O (15 mL). The solvents were removed *in vacuo* and the residue was purified by silica gel column chromatography (PE/ethyl acetate) to afford the title compound (50.0 mg, 53%) as a colorless oil.

^{1}H NMR (400 MHz, $CDCl_3$) δ 7.93–7.82 (m, 2H), 7.40–7.27 (m, 5H), 6.93–6.82 (m, 2H), 5.71 (dd, J = 9.0, 4.5 Hz, 1H), 3.84 (s, 3H), 3.70 (s, 3H), 3.07 (dd, J = 15.8, 9.0 Hz, 1H), 2.80 (dd, J = 15.8, 4.5 Hz, 1H).
^{13}C NMR (101 MHz, $CDCl_3$) δ 170.6, 166.7, 161.4, 139.6, 131.4, 128.9, 128.3, 125.9, 123.0, 115.7, 76.7 (overlapped with $CDCl_3$), 52.0, 51.8, 43.5.

HRMS (ESI): $[M + Na]^+$ calculated for $C_{18}H_{18}O_5Na$: 337.1052, found: 337.1059.

Supplementary Note 11. Experimental Procedures and Characterization for the Reaction in Fig. 3f

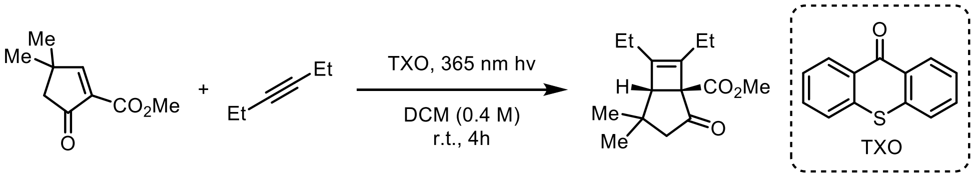

Methyl 6,7-diethyl-4,4-dimethyl-2-oxobicyclo[3.2.0]hept-6-ene-1-carboxylate

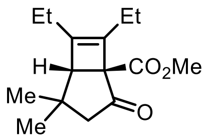

An oven-dried 10 mL Schlenk flask containing methyl 3,3-dimethyl-5-oxocyclopent-1-ene-1-carboxylate (72.1 mg, 0.43 mmol, 1.0 equiv.) and thioxanthone (TXO, 1.0 mg, 4.71 μmol, 0.01 equiv.) was evacuated and backfilled with N_2 three times, to which CH_2Cl_2 (1 mL) and 3-hexyne (98 μL, 70 mg, 0.86 mmol, 2.0 equiv.) were added via syringe. The solution was irradiated by an ultraviolet LED (365 nm, 30 W, 1 cm distance from the side) for 4 h. Then the solution was concentrated *in vacuo* and the crude was purified by silica gel column chromatography (PE/ethyl acetate) to yield methyl 6,7-diethyl-4,4-dimethyl-2-oxobicyclo[3.2.0]hept-6-ene-1-carboxylate (78.4 mg, 73%) as a colorless oil. A larger scale reaction (50 mmol) was repeated with a 92% yield.

^{1}H NMR (400 MHz, $CDCl_3$) δ 3.74 (s, 3H), 3.03 (s, 1H), 2.89 (d, J = 17.2 Hz, 1H), 2.37 (dq, J = 15.2, 7.7 Hz, 1H), 2.28–2.16 (m, 2H), 2.15–2.05 (m, 1H), 1.99 (d, J = 17.1 Hz, 1H), 1.16 (s, 3H), 1.12–1.04 (m, 6H), 1.00 (t, J = 7.6 Hz, 3H).
^{13}C NMR (101 MHz, $CDCl_3$) δ 211.09, 169.78, 148.20, 142.97, 65.43, 57.71, 52.15, 49.01, 34.87, 29.00, 24.99, 22.27, 20.57, 12.13.

HRMS (ESI): $[M + Na]^+$ calculated for $C_{15}H_{22}O_3Na$: 273.1462; found: 273.1456.

Supplementary Note 12. Web App

To make the NMR-Solver framework more accessible to the broader research community, we offer a user-friendly web application for interactive molecular structure elucidation from NMR spectra. Accessible at https://www.bohrium.com/apps/nmr-toolbox, the web-based platform provides easy access to the key functionalities of the NMR-Solver framework, with three core capabilities:

- **NMR database search**: The application performs spectrum-to-structure search by accepting a set of experimental ^{1}H and ^{13}C chemical shifts as input. It queries the built-in SimNMR-PubChem Database and returns a ranked list of candidate molecules, with support for filtering by element types.
- **Structure elucidation from NMR**: The application performs automated structure elucidation by integrating the NMR-Solver framework. Given experimental ^{1}H and ^{13}C NMR spectra, it generates a diverse set of plausible candidate molecules and ranks them based on spectral similarity and prediction confidence. Allowed element types can be specified to constrain the search space, and candidate molecules can be provided as input to guide the structure elucidation process.
- **Chemical shift prediction and spectral matching**: The application predicts ^{1}H and ^{13}C chemical shifts for given molecular structures using a deep learning model. It computes spectral similarity scores between the predicted and reference spectra, supporting structure validation and assignment.

The web interface is designed to democratize access to advanced NMR analysis, seamlessly integrating computational methods into practical chemistry workflows. It enables chemists to perform structure elucidation and spectral validation through a browser-based platform, eliminating the need for local computational infrastructure or specialized expertise.

Supplementary Tables

Supplementary Table 1. Hyperparameters for similarity metrics.

Parameter	^{1}H NMR	^{13}C NMR
**Vector Similarity**		
σ (Guassian kernel)	0.3	2
range	$[-1, 15]$	$[-10, 230]$
dimension	128	128
**Set Similarity**		
σ (Guassian kernel)	1	10

Supplementary Table 2. Hyperparameters for database index. The SimNMR-PubChem database was built with an HNSW [8] index.

D	HNSW: M	HNSW: efConstruction	HNSW: efSearch
256	32	600	2000

Supplementary Table 3. Hyperparameters for FB-MO.

Hyperparameter	Value	Description
num_search	1000	Number of candidate molecules to explore during search
num_pool	1000	Size of the candidate pool
num_filter_pair	200000	Max number of molecular pairs to consider during filtering
num_filter_mol	1000	Max number of molecules to retain after filtering

Supplementary Table 4. Comparison with current methods on the experimental dataset (stereochemistry ignored).

NMR Type	Condition	Top-1(%)	Top-3(%)	Top-10(%)	Tani[1]@1	Tani@3	Tani@10
**NMR-to-Structure [9][2]**							
^{1}H	No	0.67	0.89	1.56	0.222	0.249	0.282
	Elements[3]	—	—	—	—	—	—
	Formula[4]	7.78	10.67	12.22	0.314	0.370	0.408
^{13}C	No	2.89	6.00	8.00	0.252	0.307	0.353
	Elements	—	—	—	—	—	—
	Formula	12.89	17.33	20.22	0.366	0.418	0.470
^{1}H + ^{13}C	No	4.89	7.78	11.56	0.325	0.376	0.428
	Elements	—	—	—	—	—	—
	Formula	14.44	20.22	21.78	0.404	0.468	0.518
**NMR-Solver (Ours)**							
^{1}H	No	0.67	1.11	3.78	0.196	0.244	0.294
	Elements	3.11	7.33	12.67	0.269	0.342	0.410
	Formula	20.00	27.33	31.78	0.382	0.445	0.488
^{13}C	No	12.22	17.33	23.78	0.371	0.436	0.499
	Elements	24.00	29.56	34.67	0.488	0.540	0.603
	Formula	45.11	50.67	54.89	0.591	0.631	0.659
^{1}H + ^{13}C	No	25.56	32.89	37.33	0.524	0.594	0.643
	Elements	38.44	45.11	51.78	0.624	0.684	0.736
	Formula	**52.89**	**61.33**	**67.33**	**0.709**	**0.766**	**0.796**

[1]Tanimoto similarity.

[2]The models were reimplemented and trained using the code and simulated data provided by the original authors.

[3]The method does not support using elemental composition as an input condition.

[4]The molecular formula, in addition to being used as input for the models, was also consistently applied as a filtering criterion for the final candidate structures across all methods.

Supplementary Table 5. Comparison with current methods on the experimental dataset (stereochemistry preserved).

NMR Type	Condition	Top-1(%)	Top-3(%)	Top-10(%)	Tani@1	Tani@3	Tani@10
**NMR-to-Structure** [9]							
^{1}H	No	0.67	0.89	1.11	0.219	0.246	0.276
	Elements	—	—	—	—	—	—
	Formula	6.44	8.67	9.78	0.307	0.364	0.400
^{13}C	No	2.89	5.33	6.44	0.250	0.303	0.347
	Elements	—	—	—	—	—	—
	Formula	9.55	14.00	16.44	0.360	0.410	0.460
^{1}H + ^{13}C	No	4.00	7.11	10.00	0.320	0.371	0.419
	Elements	—	—	—	—	—	—
	Formula	10.88	16.44	17.33	0.391	0.455	0.500
**NMR-Solver (Ours)**							
^{1}H	No	0.00	1.33	2.44	0.196	0.243	0.287
	Elements	2.00	5.78	9.78	0.266	0.339	0.403
	Formula	12.00	21.78	27.33	0.350	0.414	0.456
^{13}C	No	8.89	13.33	19.11	0.364	0.426	0.488
	Elements	15.78	24.44	30.67	0.462	0.531	0.592
	Formula	30.0	42.22	45.33	0.563	0.605	0.628
^{1}H + ^{13}C	No	15.11	25.56	29.11	0.503	0.573	0.618
	Elements	22.44	34.89	40.89	0.587	0.656	0.702
	Formula	**31.56**	**47.56**	**53.78**	**0.659**	**0.720**	**0.758**

Supplementary Table 6. Comparison of NMR-Solver performance with and without reactants. [1]

NMR Type	Condition	Top-1(%)	Top-3(%)	Top-10(%)	Tani@1	Tani@3	Tani@10
**NMR-Solver (without reactants)**							
^{1}H	No	0.67	1.11	3.78	0.196	0.244	0.294
	Elements	3.11	7.33	12.67	0.269	0.342	0.410
	Formula	20.00	27.33	31.78	0.382	0.445	0.488
^{13}C	No	12.22	17.33	23.78	0.371	0.436	0.499
	Elements	24.00	29.56	34.67	0.488	0.540	0.603
	Formula	45.11	50.67	54.89	0.591	0.631	0.659
^{1}H + ^{13}C	No	25.56	32.89	37.33	0.524	0.594	0.643
	Elements	38.44	45.11	51.78	0.624	0.684	0.736
	Formula	52.89	61.33	67.33	0.709	0.766	0.796
**NMR-Solver (with reactants)**							
^{1}H	No[2]	—	—	—	—	—	—
	Elements	3.33	8.00	15.11	0.285	0.368	0.441
	Formula	25.11	34.22	40.44	0.433	0.507	0.556
^{13}C	No	—	—	—	—	—	—
	Elements	26.44	33.11	38.67	0.518	0.583	0.649
	Formula	50.89	56.89	61.56	0.647	0.687	0.718
^{1}H + ^{13}C	No	—	—	—	—	—	—
	Elements	42.89	50.67	57.78	0.664	0.726	0.779
	Formula	**60.22**	**69.11**	**76.22**	**0.767**	**0.824**	**0.858**

[1]Stereochemistry not considered.

[2]When reactants are provided, the permissible elemental composition of the products is often constrained.

Supplementary Table 7. Ablation study on the contribution of the optimization stage. [1]

NMR Type	Condition	Top-1(%)	Top-3(%)	Top-10(%)	Tani@1	Tani@3	Tani@10
**NMR-Solver (default)**							
^{1}H	No	0.67	1.11	3.78	0.196	0.244	0.294
	Elements	3.11	7.33	12.67	0.269	0.342	0.410
	Formula	20.00	27.33	31.78	0.382	0.445	0.488
^{13}C	No	12.22	17.33	23.78	0.371	0.436	0.499
	Elements	24.00	29.56	34.67	0.488	0.540	0.603
	Formula	45.11	50.67	54.89	0.591	0.631	0.659
$^{1}H + ^{13}C$	No	25.56	32.89	37.33	0.524	0.594	0.643
	Elements	38.44	45.11	51.78	0.624	0.684	0.736
	Formula	**52.89**	**61.33**	**67.33**	**0.709**	**0.766**	**0.796**
**NMR-Solver (removing target from candidates)**							
^{1}H	No	0.22 (-0.45)	0.89 (-0.22)	2.89 (-0.89)	0.192 (-0.004)	0.241 (-0.003)	0.286 (-0.008)
	Elements	2.00 (-1.11)	6.00 (-1.33)	10.22 (-2.45)	0.260 (-0.009)	0.329 (-0.013)	0.392 (-0.018)
	Formula	16.67 (-3.33)	23.33 (-4.00)	28.00 (-3.78)	0.359 (-0.023)	0.423 (-0.022)	0.469 (-0.019)
^{13}C	No	12.22 (-0.00)	16.89 (-0.44)	22.22 (-1.56)	0.369 (-0.002)	0.432 (-0.004)	0.486 (-0.013)
	Elements	22.00 (-2.00)	28.22 (-1.34)	34.00 (-0.67)	0.473 (-0.015)	0.532 (-0.008)	0.596 (-0.007)
	Formula	41.56 (-3.55)	46.89 (-3.78)	50.67 (-4.22)	0.567 (-0.024)	0.608 (-0.023)	0.634 (-0.025)
$^{1}H + ^{13}C$	No	24.60 (-0.96)	32.80 (-0.09)	36.67 (-0.66)	0.517 (-0.007)	0.592 (-0.002)	0.637 (-0.006)
	Elements	38.22 (-0.22)	43.56 (-1.55)	50.00 (-1.78)	0.623 (-0.001)	0.674 (-0.010)	0.726 (-0.010)
	Formula	**51.33 (-1.56)**	**60.00 (-1.33)**	**65.56 (-1.77)**	**0.700 (-0.009)**	**0.758 (-0.008)**	**0.786 (-0.010)**

[1]Stereochemistry not considered.

Supplementary Table 8. Performance of spectral similarity metrics under varying noise and perturbation conditions ($p = 0$). The parameter σ indicates the magnitude of spectral noise, modeled as Gaussian deviations in chemical shifts, and p denotes the probability of random peak insertion or deletion, simulating signal overlap and impurity effects. Cells shaded in gray indicate relatively noticeable poorer performance (more than 5% lower than the best performance in the row).

NMR Type	p	σ	Wasserstein	Vector	Set
		Top-1 Recall (%)			
^1H	0.0	0.0	100	100	100
		0.1	75.2	55.8	76.9
		0.2	27.4	7.4	31.1
		0.3	5.7	0.9	7.9
^{13}C	0.0	0.0	100	100	100
		0.1	86.3	80.2	87.9
		0.2	47.6	16.8	50.9
		0.3	16.0	2.4	17.4
^1H + ^{13}C	0.0	0.0	100	100	100
		0.1	96.7	93.3	95.5
		0.2	85.2	52.9	86.2
		0.3	56.6	13.5	57.7
		Top-10 Recall (%)			
^1H	0.0	0.0	100	100	100
		0.1	91.9	81.3	92.8
		0.2	50.9	20.4	53.0
		0.3	13.2	3.0	15.4
^{13}C	0.0	0.0	100	100	100
		0.1	99.2	96.5	99.0
		0.2	70.1	38.3	70.7
		0.3	28.3	7.3	28.4
^1H + ^{13}C	0.0	0.0	100	100	100
		0.1	100	99.5	99.9
		0.2	97.0	80.0	95.8
		0.3	71.0	30.8	68.5

Supplementary Table 9. Performance of spectral similarity metrics under varying noise and perturbation conditions ($p = 0.2$). The parameter σ indicates the magnitude of spectral noise, modeled as Gaussian deviations in chemical shifts, and p denotes the probability of random peak insertion or deletion, simulating signal overlap and impurity effects. Cells shaded in gray indicate relatively noticeable poorer performance (more than 5% lower than the best performance in the row).

NMR Type	p	σ	Wasserstein	Vector	Set
Top-1 Recall (%)					
^{1}H	0.2	0.0	88.3	95.5	88.3
		0.1	63.0	52.9	67.7
		0.2	22.5	7.0	27.3
		0.3	4.6	0.8	7.0
^{13}C	0.2	0.0	83.9	99.1	90.7
		0.1	70.9	77.1	79.7
		0.2	39.0	16.3	45.9
		0.3	13.2	2.3	15.4
^{1}H + ^{13}C	0.2	0.0	90.3	100	98.3
		0.1	84.0	92.6	93.3
		0.2	72.2	51.9	83.3
		0.3	47.6	12.9	55.0
Top-10 Recall (%)					
^{1}H	0.2	0.0	91.6	98.4	94.0
		0.1	80.0	78.0	86.3
		0.2	42.9	19.3	49.0
		0.3	11.0	2.7	14.1
^{13}C	0.2	0.0	87.8	100	97.5
		0.1	85.5	95.0	96.0
		0.2	59.3	36.8	67.5
		0.3	24.1	7.0	26.5
^{1}H + ^{13}C	0.2	0.0	95.4	100	99.9
		0.1	93.0	99.5	99.7
		0.2	87.9	78.5	95.2
		0.3	63.1	30.1	66.9

Supplementary Table 10. Comparison of NMR-Solver performance with or without multiplicity information. Cells shaded in gray indicate improved performance over using chemical shifts alone.

NMR Type	Condition	Top-1(%)	Top-3(%)	Top-10(%)	Tani@1	Tani@3	Tani@10
**NMR-Solver (chemical shifts only)**							
^1H	No	0.67	1.11	3.78	0.196	0.244	0.294
	Elements	3.11	7.33	12.67	0.269	0.342	0.410
	Formula	20.00	27.33	31.78	0.382	0.445	0.488
**NMR-Solver (multiplicity included)**							
^1H	No	1.60	2.29	5.03	0.224	0.266	0.319
	Elements	3.78	8.22	11.56	0.293	0.353	0.420
	Formula	18.44	25.78	32.00	0.382	0.449	0.493

Supplementary Table 11. Allowed cleavage bond pairings in FB-MO. For each query fragment cleavage bond, the table lists valid complementary cleavage bonds that satisfy the local environment preservation constraint. Here, **C** denotes a carbon atom, and **D** denotes a non-carbon atom (e.g., N, O, S). $\mathbf{A}_1$, $\mathbf{A}_2$, and $\mathbf{A}_3$ represent any chemical atom. Halogens (F, Cl, Br, I) are treated as a single element class for pairing purposes, reflecting their similar bonding behavior in fragment recombination.

Query Cleavage Bond	Allowed Complementary Cleavage Bonds
$\mathrm{C{-}A_1}$	$\mathrm{A_1{-}A_2}$
$\mathrm{C{=}A_1}$	$\mathrm{A_1{=}A_2}$
$\mathrm{C{\equiv}A_1}$	$\mathrm{A_1{\equiv}A_2}$
$\mathrm{D{-}A_3}$	$\mathrm{A_1{-}A_2}$
$\mathrm{D{=}A_3}$	$\mathrm{A_1{=}A_2}$
$\mathrm{D{\equiv}A_3}$	$\mathrm{A_1{\equiv}A_2}$

Supplementary Figures

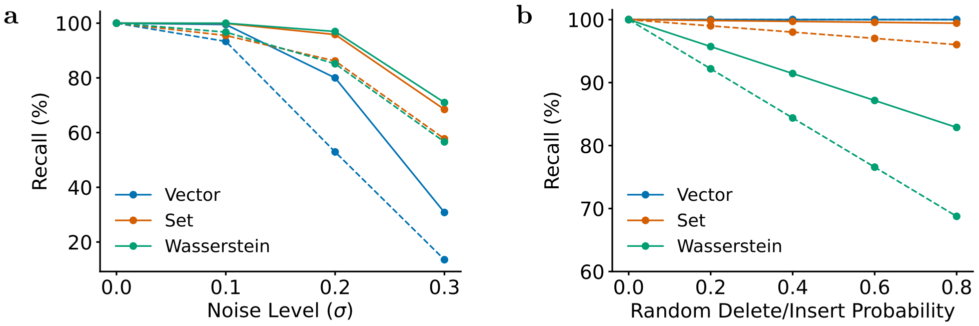

Supplementary Figure 1. Comparison of spectral similarity metrics under varying noise and perturbation conditions. **a.** Recall vs. spectral noise level, where chemical shift deviations are modeled as Gaussian noise with a standard deviation of σ ppm for ^{1}H NMR and 10σ ppm for ^{13}C NMR. **b.** Recall vs. random peak deletion/insertion probability, simulating signal overlap and impurity effects. Dashed lines indicate top-1 recall, while solid lines indicate top-10 recall. Analyses and statistical summaries were performed on 1000 molecules.

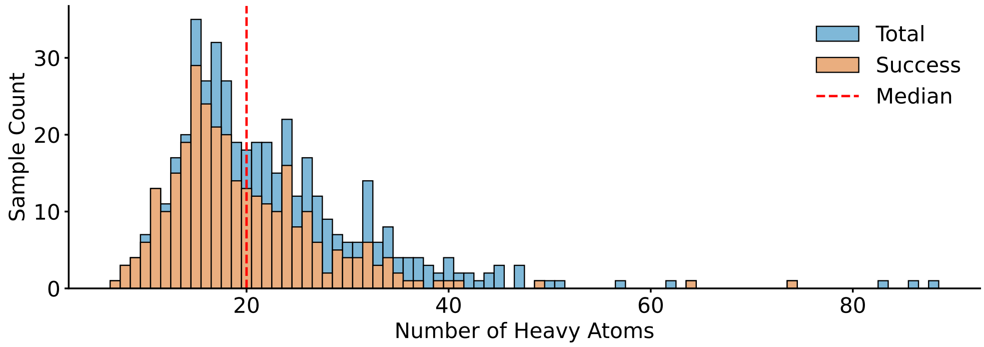

Supplementary Figure 2. Atom count distribution of the 450 molecules in the literature dataset. Correct prediction means the ground truth appears in the top 10 solutions.

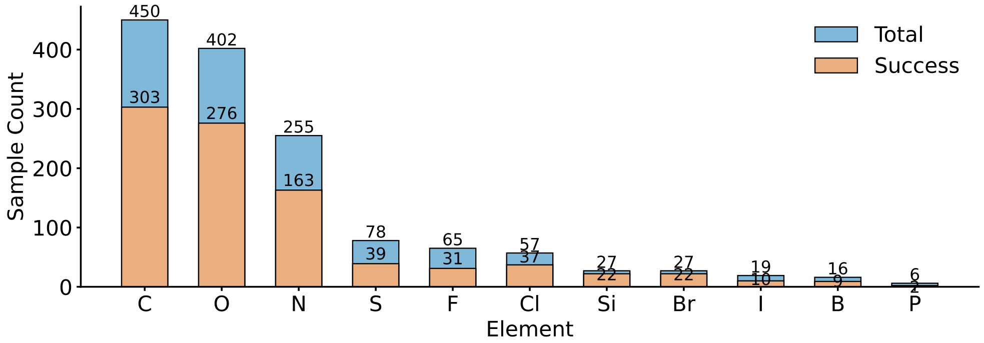

Supplementary Figure 3. Element statistics of the 450 molecules in the literature dataset. Correct prediction means the ground truth appears in the top 10 solutions.

Rank	Retrieved Candidates	Optimized Structures
**1**	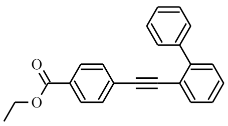 **Score: 0.953**	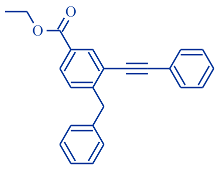 **Score: 0.997**
**2**	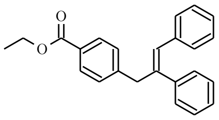 **Score: 0.936**	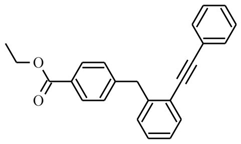 **Score: 0.996**
**3**	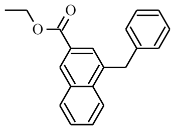 **Score: 0.930**	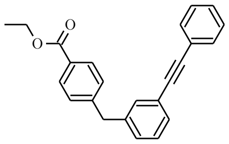 **Score: 0.991**
**4**	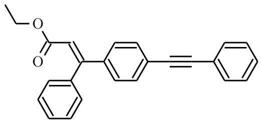 **Score: 0.918**	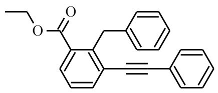 **Score: 0.991**
**5**	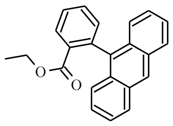 **Score: 0.917**	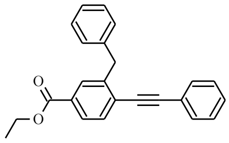 **Score: 0.991**

Supplementary Figure 4. NMR-Solver's Predicted Structures for the Case in Fig. 3a.

Rank	Retrieved Candidates	Optimized Structures
**1**	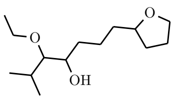 **Score: 0.933**	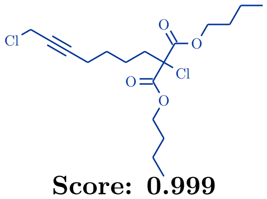 **Score: 0.999**
**2**	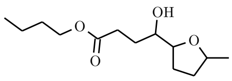 **Score: 0.931**	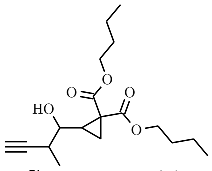 **Score: 0.977**
**3**	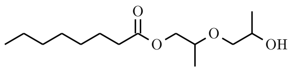 **Score: 0.923**	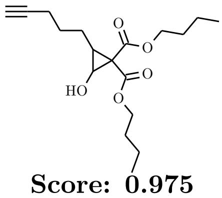 **Score: 0.975**
**4**	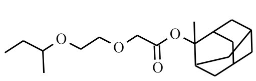 **Score: 0.922**	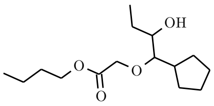 **Score: 0.969**
**5**	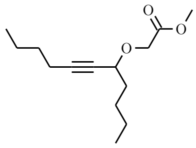 **Score: 0.922**	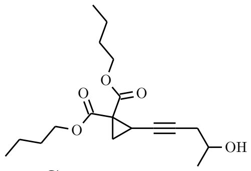 **Score: 0.968**

Supplementary Figure 5. NMR-Solver's Predicted Structures for the Case in Fig. 3b.

Rank	Retrieved Candidates	Optimized Structures
**1**	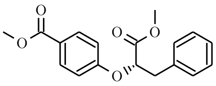<br>**Score: 0.965**	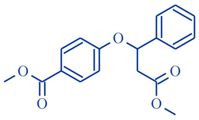<br>**Score: 0.980**
**2**	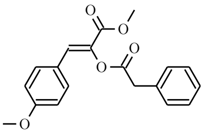<br>**Score: 0.924**	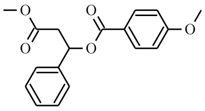<br>**Score: 0.971**
**3**	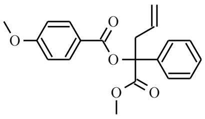<br>**Score: 0.917**	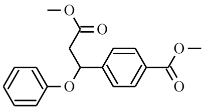<br>**Score: 0.969**
**4**	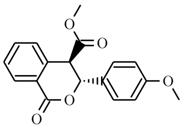<br>**Score: 0.898**	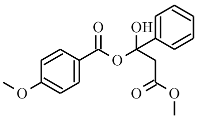<br>**Score: 0.965**
**5**	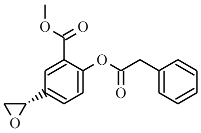<br>**Score: 0.890**	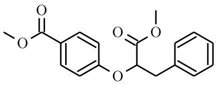<br>**Score: 0.965**

Supplementary Figure 6. NMR-Solver's Predicted Structures for the Case in Fig. 3c.

Rank	Retrieved Candidates	Optimized Structures	
1	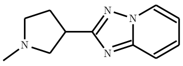 Score: 0.863	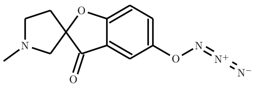 Score: 0.978	(Excluded by formula)
2	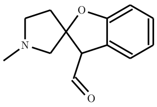 Score: 0.863	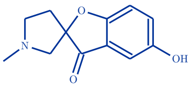 Score: 0.972	
3	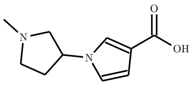 Score: 0.858	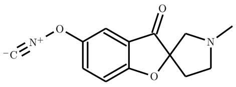 Score: 0.966	
4	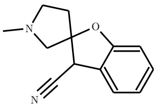 Score: 0.841	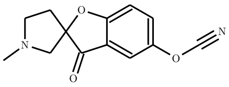 Score: 0.961	
5	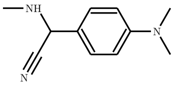 Score: 0.832	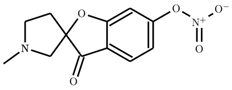 Score: 0.960	

Supplementary Figure 7. NMR-Solver's Predicted Structures for the Case in Fig. 3d.

Rank	Retrieved Candidates	Optimized Structures
**1**	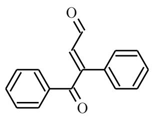 **Score: 0.981**	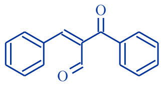 **Score: 0.993**
**2**	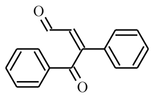 **Score: 0.981**	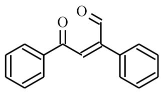 **Score: 0.986**
**3**	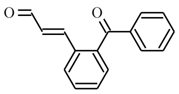 **Score: 0.955**	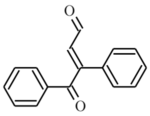 **Score: 0.981**
**4**	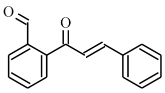 **Score: 0.955**	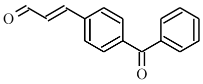 **Score: 0.981**
**5**	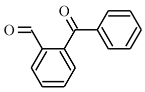 **Score: 0.937**	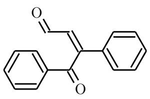 **Score: 0.981**

Supplementary Figure 8. NMR-Solver's Predicted Structures for the Case in Fig. 3e.

Rank	Retrieved Candidates	Optimized Structures
1	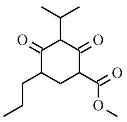 **Score: 0.927**	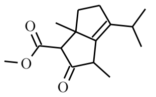 **Score: 0.975**
2	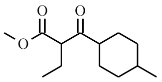 **Score: 0.918**	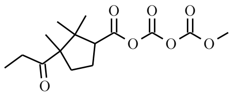 **Score: 0.970**
3	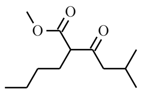 **Score: 0.913**	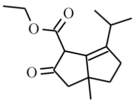 **Score: 0.966**
4	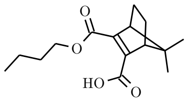 **Score: 0.910**	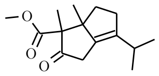 **Score: 0.962**
5	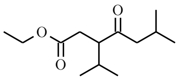 **Score: 0.894**	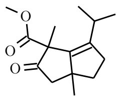 **Score: 0.960**

Supplementary Figure 9. NMR-Solver's Predicted Structures for the Case in Fig. 3f.

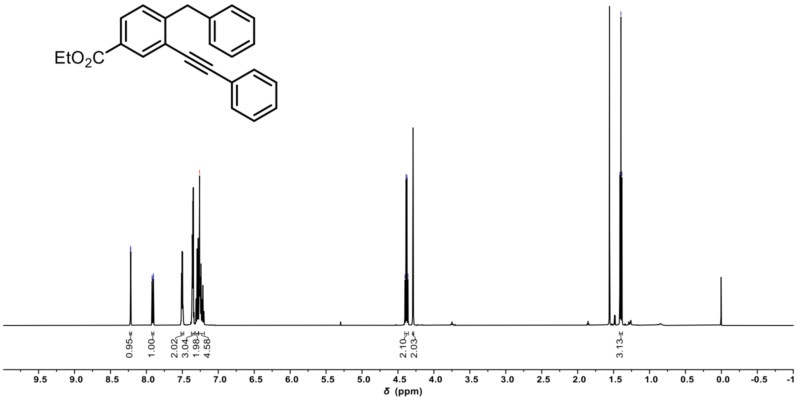

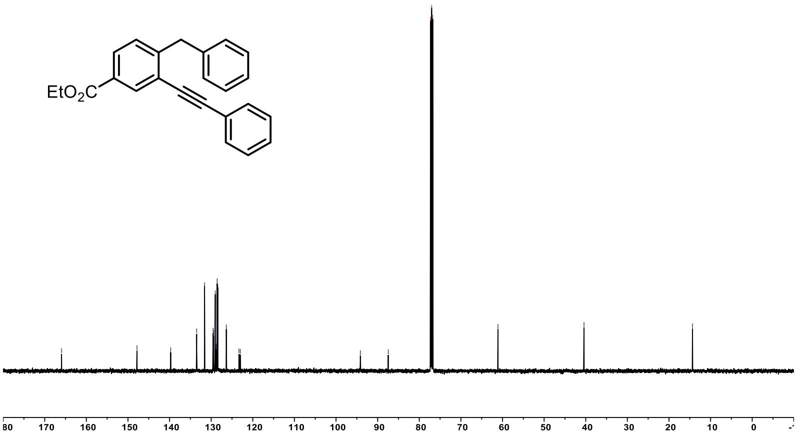

Supplementary Figure 10. ^{1}H and ^{13}C NMR spectra of ethyl 4-benzyl-3-(phenylethynyl)benzoate.

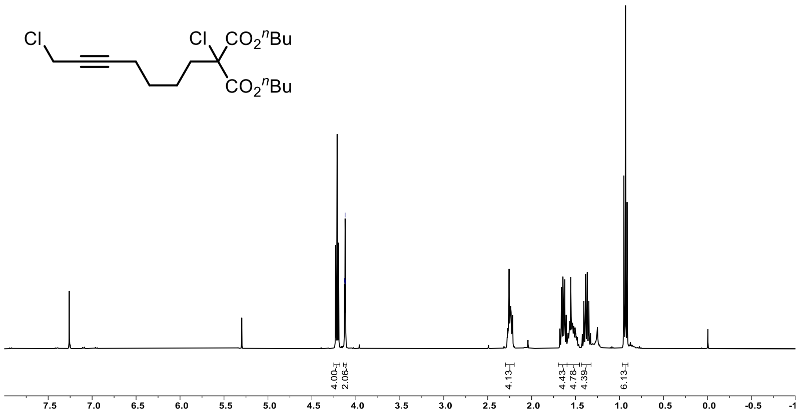

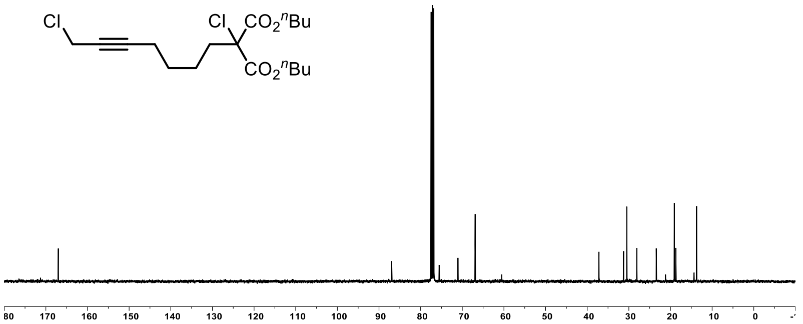

Supplementary Figure 11. ^{1}H and ^{13}C NMR spectra of dibutyl 2-chloro-2-(7-chlorohept-5-yn-1-yl)malonate.

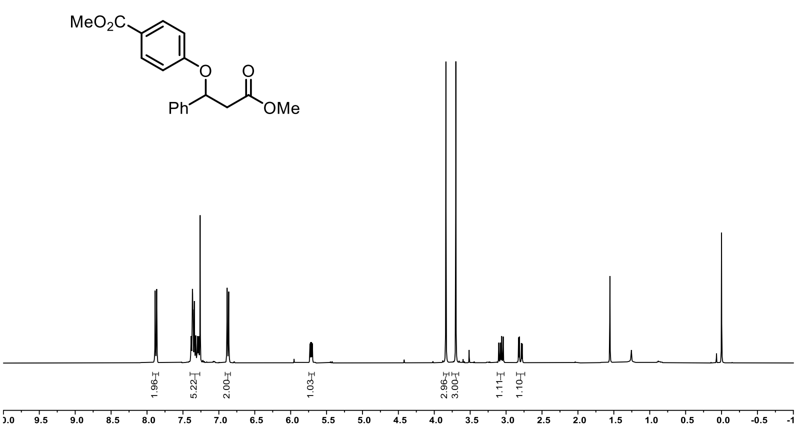

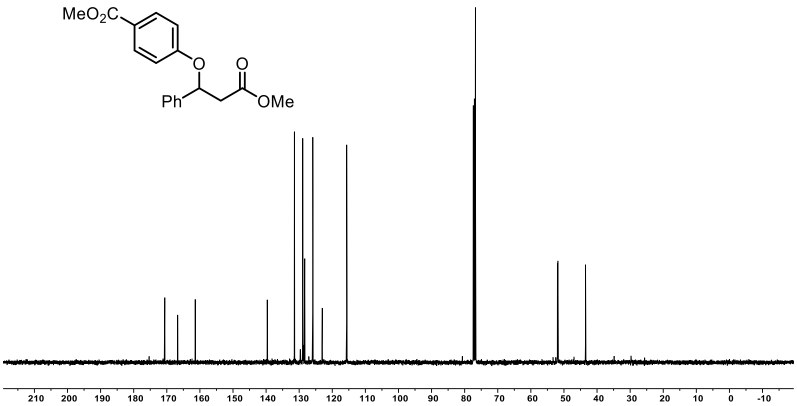

Supplementary Figure 12. ^{1}**H and** ^{13}**C NMR spectra of methyl 4-(3-methoxy-3-oxo-1-phenylpropoxy)benzoate.**

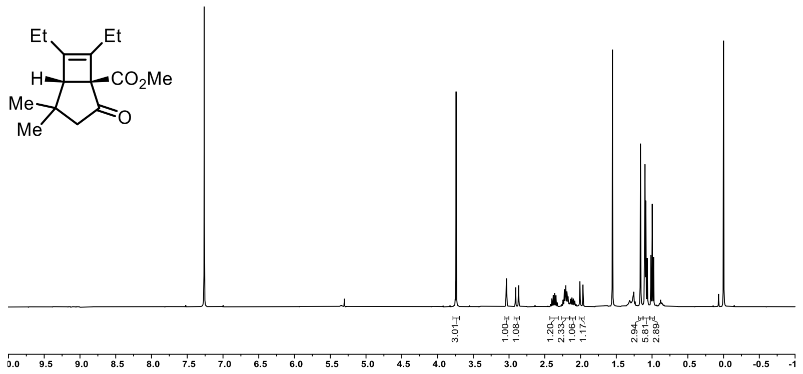

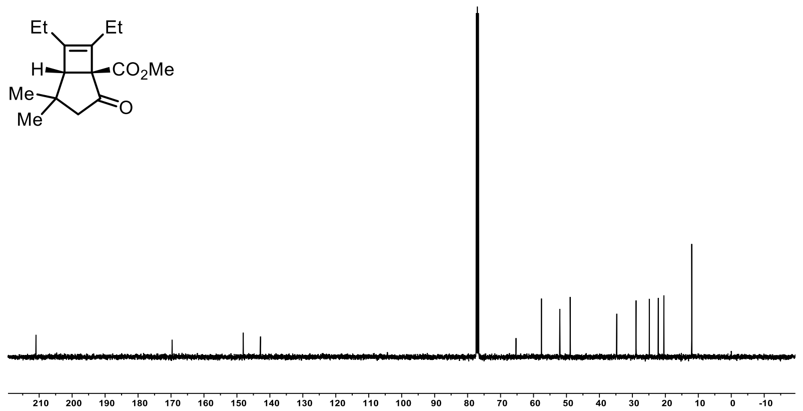

Supplementary Figure 13. ^{1}**H and** ^{13}**C NMR spectra of methyl 6,7-diethyl-4,4-dimethyl-2-oxobicyclo[3.2.0]hept-6-ene-1-carboxylate.**

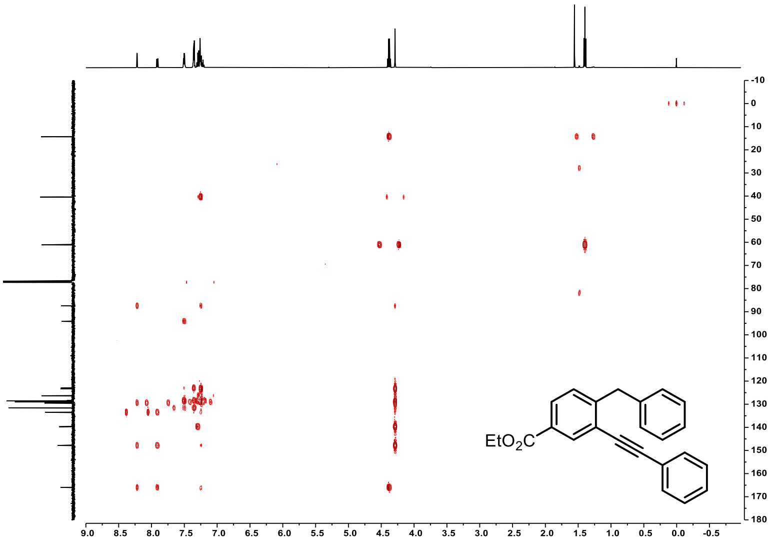

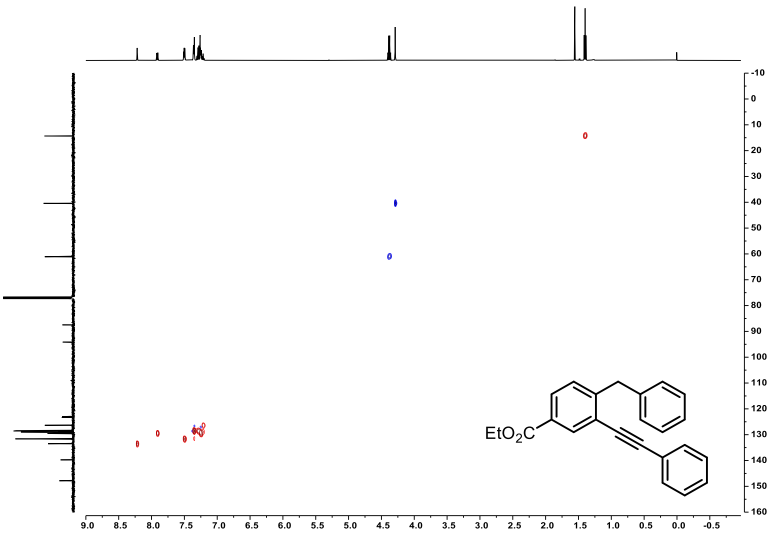

Supplementary Figure 14. ^{1}H-^{13}C HMBC and HSQC spectra of ethyl 4-benzyl-3-(phenylethynyl)benzoate. (500 MHz, $CDCl_3$).

Supplementary References

[1] Tanimoto, T. T. An elementary mathematical theory of classification and prediction (1958).

[2] Bajusz, D., Rácz, A. & Héberger, K. Why is tanimoto index an appropriate choice for fingerprint-based similarity calculations? *Journal of cheminformatics* **7**, 20 (2015).

[3] Morgan, H. L. The generation of a unique machine description for chemical structures-a technique developed at chemical abstracts service. *Journal of chemical documentation* **5**, 107–113 (1965).

[4] Rogers, D. & Hahn, M. Extended-connectivity fingerprints. *Journal of chemical information and modeling* **50**, 742–754 (2010).

[5] Rubner, Y., Tomasi, C. & Guibas, L. J. The earth mover's distance as a metric for image retrieval. *International journal of computer vision* **40**, 99–121 (2000).

[6] Fu, Y. *et al.* Photocatalyzed dehydroxylative amination of phenols: A ring-expansion approach for medium-sized benzolactams. *Organic Letters* **23**, 8317–8321 (2021).

[7] Cheng, D., Yu, C., Pu, Y. & Xu, X. DDQ-mediated oxidative coupling reaction of N, N-dimethyl enaminones with cycloheptatriene. *Tetrahedron Letters* **90**, 153609 (2022).

[8] Malkov, Y. A. & Yashunin, D. A. Efficient and robust approximate nearest neighbor search using hierarchical navigable small world graphs. *IEEE transactions on pattern analysis and machine intelligence* **42**, 824–836 (2018).

[9] Alberts, M., Schilter, O., Zipoli, F., Hartrampf, N. & Laino, T. Unraveling molecular structure: A multimodal spectroscopic dataset for chemistry. *Advances in Neural Information Processing Systems* **37**, 125780–125808 (2024).
