# Supplementary material for: NMR-Solver: automated structure elucidation via large-scale spectral matching and physics-guided fragment optimization
Source: Nat Commun. 2026 Apr 2;17:4740. doi: 10.1038/s41467-026-71315-0 (PMC13216333; doi:10.1038/s41467-026-71315-0)
Supplement: Supplementary file 2 — Reporting Summary [file 41467_2026_71315_MOESM2_ESM.pdf]

nature portfolio

Corresponding author(s): Weinan E

Last updated by author(s): Feb 2, 2026

Reporting Summary

Nature Portfolio wishes to improve the reproducibility of the work that we publish. This form provides structure for consistency and transparency in reporting. For further information on Nature Portfolio policies, see our Editorial Policies and the Editorial Policy Checklist.

Statistics

For all statistical analyses, confirm that the following items are present in the figure legend, table legend, main text, or Methods section.

n/a	Confirmed	
☐	☒	The exact sample size (n) for each experimental group/condition, given as a discrete number and unit of measurement
☐	☒	A statement on whether measurements were taken from distinct samples or whether the same sample was measured repeatedly
☒	☐	The statistical test(s) used AND whether they are one- or two-sided<br>*Only common tests should be described solely by name; describe more complex techniques in the Methods section.*
☐	☒	A description of all covariates tested
☒	☐	A description of any assumptions or corrections, such as tests of normality and adjustment for multiple comparisons
☐	☒	A full description of the statistical parameters including central tendency (e.g. means) or other basic estimates (e.g. regression coefficient) AND variation (e.g. standard deviation) or associated estimates of uncertainty (e.g. confidence intervals)
☒	☐	For null hypothesis testing, the test statistic (e.g. F, t, r) with confidence intervals, effect sizes, degrees of freedom and P value noted<br>*Give P values as exact values whenever suitable.*
☒	☐	For Bayesian analysis, information on the choice of priors and Markov chain Monte Carlo settings
☒	☐	For hierarchical and complex designs, identification of the appropriate level for tests and full reporting of outcomes
☒	☐	Estimates of effect sizes (e.g. Cohen's d, Pearson's r), indicating how they were calculated

Our web collection on statistics for biologists contains articles on many of the points above.

Software and code

Policy information about availability of computer code

Data collection	Experimental NMR spectra were collected using a Bruker Avance III 400 MHz spectrometer at room temperature. Raw data were processed using TopSpin (version 4.1.7).
Data analysis	- NMR-Solver was developed using Python 3.10 and built mainly over PyTorch (version 2.5.1), NumPy (version 2.3.5), Pandas (version 2.3.3), SciPy (version 1.16.3), FAISS (version 1.5.3) and RDKit (version 2025.3.3).<br>- Experimental NMR spectra were additionally processed using MNova (version 14.2.0).<br>- Data visualization was performed using Matplotlib (version 3.10.0).<br>- Molecules were processed and visualized using ChemDraw (version 22.0).<br><br>The source code for NMR-Solver is available on GitHub (https://github.com/YongqiJin/NMR-Solver) with an associated ReadMe file and available on Zenodo at https://doi.org/10.5281/zenodo.18450044 (version 1.0).

For manuscripts utilizing custom algorithms or software that are central to the research but not yet described in published literature, software must be made available to editors and reviewers. We strongly encourage code deposition in a community repository (e.g. GitHub). See the Nature Portfolio guidelines for submitting code & software for further information.

Data

Policy information about availability of data

All manuscripts must include a data availability statement. This statement should provide the following information, where applicable:

- Accession codes, unique identifiers, or web links for publicly available datasets
- A description of any restrictions on data availability
- For clinical datasets or third party data, please ensure that the statement adheres to our policy

All processed NMR datasets used in this study are available via Zenodo at https://doi.org/10.5281/zenodo.16952024. The processed dataset of the SimNMR-PubChem Database is available on Hugging Face at https://huggingface.co/datasets/yqj01/SimNMR-PubChem. Original NMR spectra are publicly available at PubChem (https://pubchem.ncbi.nlm.nih.gov).

Research involving human participants, their data, or biological material

Policy information about studies with human participants or human data. See also policy information about sex, gender (identity/presentation), and sexual orientation and race, ethnicity and racism.

Reporting on sex and gender	This study did not involve human participants, human data, or human biological material.
Reporting on race, ethnicity, or other socially relevant groupings	Not applicable as no human participants were involved.
Population characteristics	Not applicable as no human participants were involved.
Recruitment	Not applicable as no human participants were involved.
Ethics oversight	Not applicable as no human participants were involved.

Note that full information on the approval of the study protocol must also be provided in the manuscript.

Field-specific reporting

Please select the one below that is the best fit for your research. If you are not sure, read the appropriate sections before making your selection.

☒ Life sciences ☐ Behavioural & social sciences ☐ Ecological, evolutionary & environmental sciences

For a reference copy of the document with all sections, see nature.com/documents/nr-reporting-summary-flat.pdf

Life sciences study design

All studies must disclose on these points even when the disclosure is negative.

Sample size	Sample sizes were determined by the availability of curated data in each dataset and computational resource considerations. The simulated dataset consisted of 1,000 randomly sampled molecules, and the literature-derived dataset contained 450 curated examples. No formal statistical power calculation was performed. These sample sizes were sufficient to enable stable evaluation in this study.
Data exclusions	Data exclusions were applied during preprocessing of the literature-derived dataset. NMR spectra with incomplete information or obvious parsing errors were excluded using predefined, automated filtering criteria. No data were excluded after model training or evaluation.
Replication	Computational experiments were deterministic with fixed random seeds. NMR spectra were acquired once per compound; no biological or technical replicates were performed.
Randomization	Randomization was applied only during the sampling of test molecules for the simulated dataset. No additional random allocation of samples was required, as the study does not involve experimental interventions or treatment groups.
Blinding	Blinding was not applicable to this study, as all data processing, algorithm execution, and evaluation were conducted through fully automated and deterministic computational pipelines, without human involvement in group assignment or outcome assessment.

Reporting for specific materials, systems and methods

We require information from authors about some types of materials, experimental systems and methods used in many studies. Here, indicate whether each material, system or method listed is relevant to your study. If you are not sure if a list item applies to your research, read the appropriate section before selecting a response.

Materials & experimental systems

n/a	Involved in the study
☒	☐ Antibodies
☒	☐ Eukaryotic cell lines
☒	☐ Palaeontology and archaeology
☒	☐ Animals and other organisms
☒	☐ Clinical data
☒	☐ Dual use research of concern
☒	☐ Plants

Methods

n/a	Involved in the study
☒	☐ ChIP-seq
☒	☐ Flow cytometry
☒	☐ MRI-based neuroimaging

Plants

Seed stocks

Report on the source of all seed stocks or other plant material used. If applicable, state the seed stock centre and catalogue number. If plant specimens were collected from the field, describe the collection location, date and sampling procedures.

Novel plant genotypes

Describe the methods by which all novel plant genotypes were produced. This includes those generated by transgenic approaches, gene editing, chemical/radiation-based mutagenesis and hybridization. For transgenic lines, describe the transformation method, the number of independent lines analyzed and the generation upon which experiments were performed. For gene-edited lines, describe the editor used, the endogenous sequence targeted for editing, the targeting guide RNA sequence (if applicable) and how the editor was applied.

Authentication

Describe any authentication procedures for each seed stock used or novel genotype generated. Describe any experiments used to assess the effect of a mutation and, where applicable, how potential secondary effects (e.g. second site T-DNA insertions, mosiacism, off-target gene editing) were examined.
